# A Comparative Study on the Management of Acute Otitis Externa Using Hydroxylated Polyvinyl Acetate Ichthammol Glycerine Wick Versus Cotton Ichthammol Glycerine Wick

**DOI:** 10.7759/cureus.65310

**Published:** 2024-07-24

**Authors:** Shyamala K, Mohana Karthikeyan S, Sheetal K

**Affiliations:** 1 Otolaryngology and Head and Neck Surgery, Karpaga Vinayaga Institute of Medical Sciences and Research Center, Maduranthakam, IND

**Keywords:** ichthammol glycerine ig, visual analog scale (vas), external auditory canal eac ., hydroxylated polyvinyl acetate pva, acute otitis externa aoe

## Abstract

Introduction: Acute otitis externa is a localized inflammation of the skin of the external auditory meatus. It is characterized by pain, edema, erythema, and itchy discomfort. Treatment includes topical and oral antibiotics, analgesics, steroids, and anti-inflammatory medication for the ear. Aural medicated wicks are used to reduce edema and pain.

Aim: To compare the clinical outcome of hydroxylated polyvinyl acetate ichthammol glycerine wick versus cotton ichthammol glycerine wick used in the treatment of acute otitis externa.

Materials and methods: It is a six-month observational study with 120 patients. The patients in this study were grouped into two groups with hydroxylated polyvinyl acetate and cotton wick, respectively. Pain was assessed using the VAS score before and after three days of treatment of acute otitis externa.

Result: Group B (patient treated with cotton ichthammol glycerine wick) had significant improvement in the pain score on days 2 and 3 compared to group A, with a significant p-value of <0.001.

Conclusion: During the second visit (on day 2), the cotton ear wick was significantly better in terms of otalgia when compared with the hydroxylated polyvinyl acetate. The cotton wick group showed better and faster recovery in terms of pain and edema compared to the polyvinyl alcohol (PVA) groups.

## Introduction

Acute otitis externa is a localized inflammation of the skin of the external auditory meatus. It is characterized by pain, edema, erythema, and itchy discomfort. Predisposing factors are local trauma and the use of external objects (ear buds, ear plugs, sharp objects, fingernails) which make the canal epithelium susceptible to infections [1,2]. Common pathogens include *Staphylococcus aureus*, pseudomonas, anaerobes, and gram-negative organisms [3]. Treatment includes topical and oral antibiotics, analgesics, steroids, and anti-inflammatory medication for the ear. Aural medicated wicks are used to reduce edema and pain. Various types of medicated wicks have been tried for the management of acute otitis externa with varying results, but none have been standardized. The role of polyvinyl acetate and its uses in the nose as a compressive nasal pack postoperatively have been known for years in otolaryngology practice. But their use in the ear has not been documented previously. The evaluation of hydroxylated polyvinyl acetate medicated ear wick in patients with acute otitis externa has been reported in a limited amount of literature only [1,4]. As a result, the present study aimed to compare the outcome of hydroxylated polyvinyl acetate wick with cotton medicated ear wick in patients with mild to moderate acute otitis externa.

## Materials and methods

A total of 120 patients were enrolled in an observational study done between March 2023 and August 2023 at the Karpaga Vinayaga Institute of Medical Sciences and Research Center, Maduranthakam, India. These patients were clinically diagnosed as acute otitis externa cases. Written informed consent was obtained from each patient after discussing the procedure in full detail and getting approval from the Ethical Committee. All patients included in this study were cases of acute otitis externa and acute earache, with the use of external objects (ear buds, ear plugs, sharp objects, fingernails, ear aids) in the age groups of 18 to 80 years. Those who have chronic suppurative otitis media, acute suppurative otitis media, otomycosis, malignant otitis externa, tumors of the external auditory canal (EAC), congenital or obstructive pathology of the EAC, symptoms lasting more than three weeks, and uncontrolled diabetes mellitus with symptoms of acute ear ache were excluded.

Patients were enrolled in this study on the basis of the inclusion and exclusion criteria, and if there was any evidence of discharge from the middle ear, then ear packing was avoided in those cases. The patients were randomly divided into two groups, in which one group (group A) was packed with hydroxylated polyvinyl acetate and the other group (group B) with cotton wick. All patients with acute otitis externa were managed with oral analgesics, topical antibiotic ear drops, and an ichthammol glycerine ear pack, either with hydroxylated polyvinyl acetate or cotton wick. The pain score was assessed and recorded before packing the ear with the help of a senturia grading (Table 1) and a numerical rating scale. After which, patients were reassessed on the subsequent days on days 1, 2, and 3 and the pain scoring was recorded on the same scale.

**Table 1:** Patients graded according to Senturia classification EAM: external auditory meatus; TM: tympanic membrane

Senturia grading	Severity of symptoms	Otoscopy
S1	Mild	Edema, dry scaly skin
S2a	Mild to moderate	Edema, redness, clear wet scales, odorless secretions
S2b	Moderate	Edema, narrowing of EAM, sero-purulent secretions, TM obscured partially, peri auricular swelling
S2c	Severe	Near total closure of EAM, TM not visible, thick, wet scaly amalgam with purulent secretions, swelling over the mastoid process

## Results

**Figure 1:**
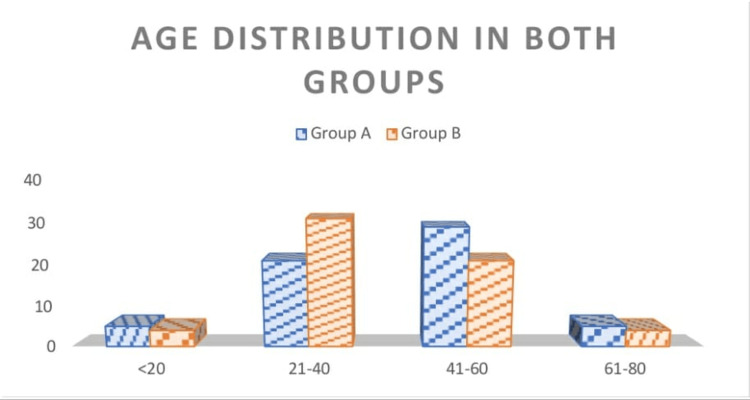
Age description

**Figure 2:**
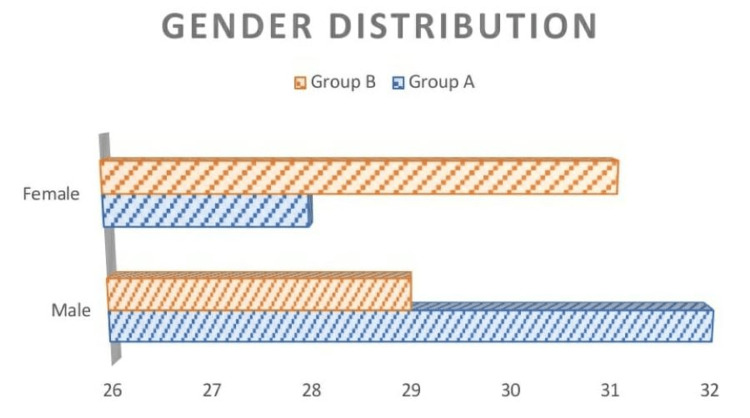
Gender description

**Figure 3:**
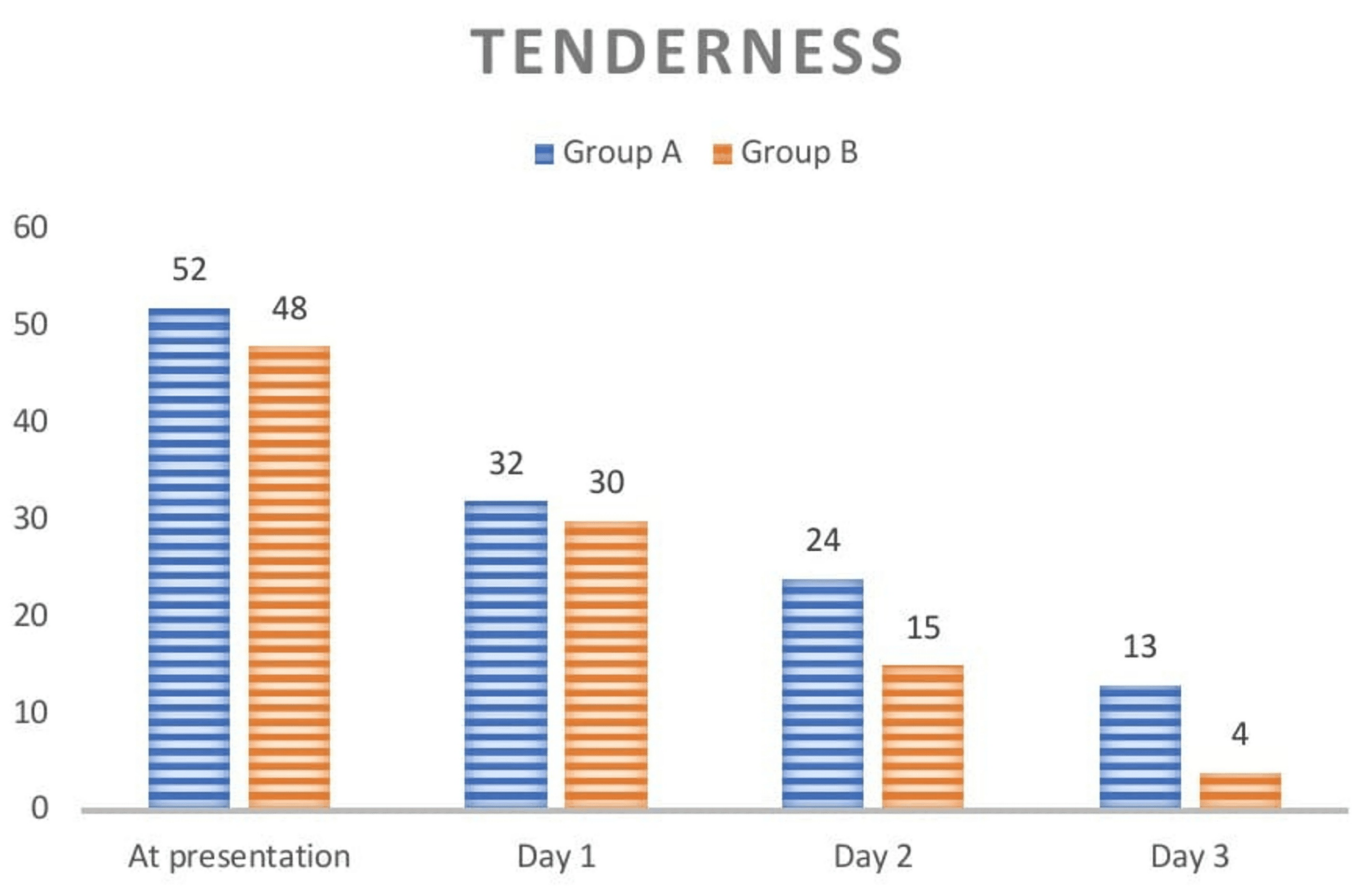
Comparison of symptoms of acute otitis externa between two groups

**Figure 4:**
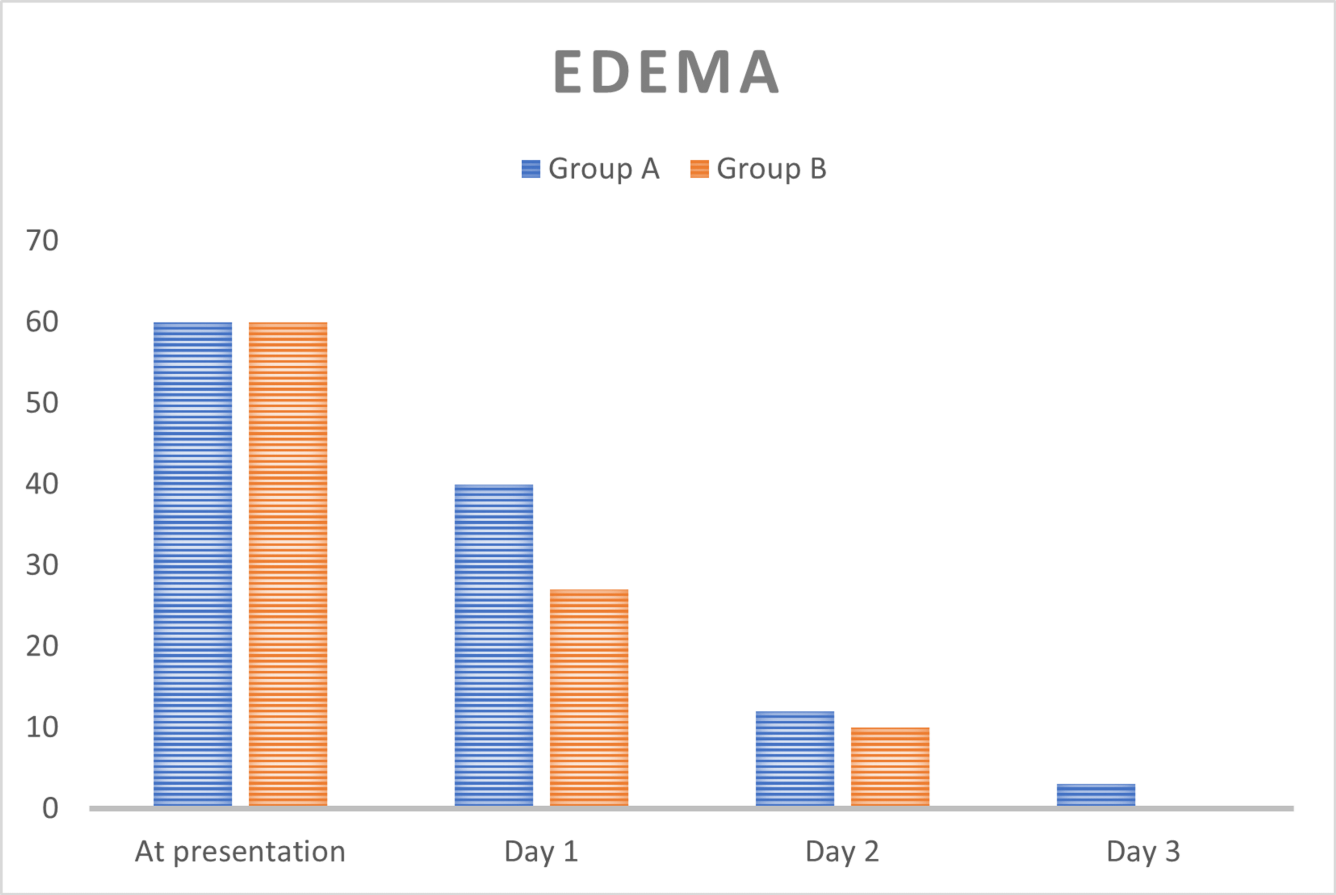
Comparison of edema between two groups

**Figure 5:**
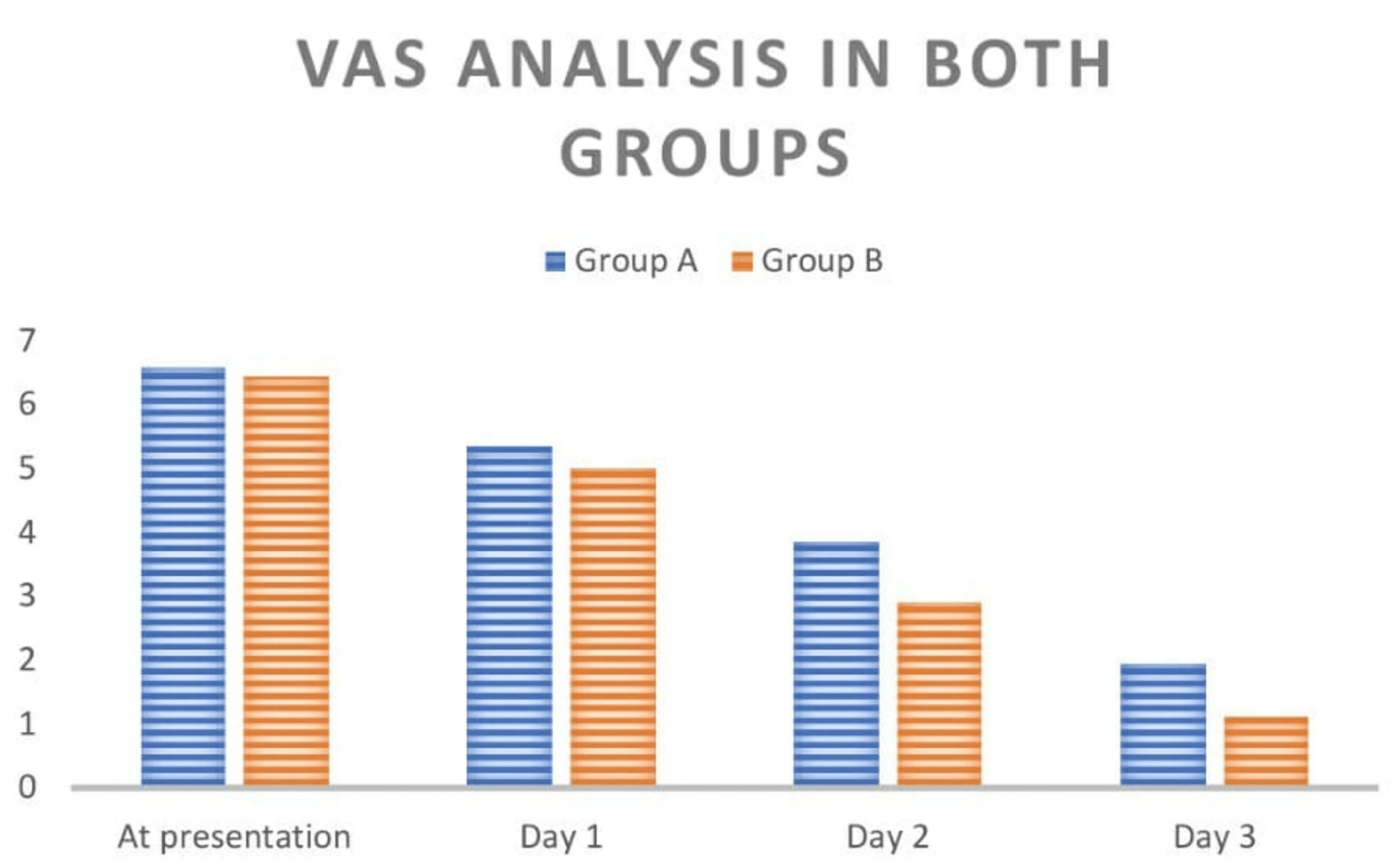
Comparison of visual analog score between two groups VAS: visual analog score

In total, 120 patients were included in this study. They were randomly assigned into two groups: one with a hydroxylated polyvinyl acetate pack and the other with a cotton wick pack, each with 60 patients, respectively. The population included in this were between the ages of 18 and 80, with a mean age of 38.94 and a standard deviation of 13.81. The age distribution in both groups is given in (Table 2, Figure 1) with the mean ages in groups A and B being 41.80 ± 13.93 and 36.07 ± 13.07, respectively. The gender distribution in this study is given in (Table 3, Figure 2) with a slight male and female preponderance in groups A and B, respectively. In regards to tenderness, there were statistically significant differences between the hydroxylated polyvinyl acetate pack and the cotton ichthammol glycerine wick pack, with a p-value of <0.001 (Table 4, Figure 3). There is a statistically significant difference in relation to the edema in both groups, with a p-value of <0.001 (Table 5, Figure 4). The pain score in relation to both groups showed a significant difference on day 2 and day 3 post-treatment, with a p-value of <0.001 (Table 6, Figure 5). In the group with the hydroxylated polyvinyl acetate pack, some patients came with increased pain and were diagnosed as furunculosis on day 2 and some on day 3. This cotton pack is better than the hydroxylated polyvinyl acetate pack.

**Table 2:** Age distribution

Age	Group A	%	Group B	%
<20	5	8.33	4	6.67
21-40	21	35	31	51.67
41-60	29	48.33	21	35
61-80	5	8.33	4	6.67

**Table 3:** Gender distribution

	Group A	%	Group B	%
Male	32	53.33	29	48.33
Female	28	46.67	31	51.67
Total	60		60	

**Table 4:** Comparison of symptoms of acute otitis externa among groups

Tenderness	Group A		Group B	
	Present	Absent	Present	Absent
At presentation	52 (86.67)	8 (13.3)	48 (80)	12 (20)
Day 1	32 (53.3)	28 (46.67)	30 (50)	30 (50)
Day 2	24 (40)	36 (60)	15 (25)	45 (75)
Day 3	13 (21.67)	47 (78.3)	4 (6.67)	56 (93.3)

**Table 5:** Comparison of edema between two groups

Edema	Group A			Group B		
	Absent	<50%	>50%	Absent	<50%	>50%
At presentation	0	0	60 (100)	0	0	60 (100)
Day 1	4 (6.67)	16 (26.67)	40 (66.67)	7 (11.67)	26 (43.3)	27 (45)
Day 2	22 (36.67)	26 (43.33)	12 (20)	19 (31.67)	31 (51.67)	10 (16.67)
Day 3	43 (71.67)	14 (23.3)	3 (5)	52 (86.67)	8 (13.3)	0

**Table 6:** Comparison of visual analog score between two groups NS: not significant; S: significant

Visual analog score	Group A	Group B	p-value
At presentation	6.59 ± 1.45	6.46 ± 1.49	0.569 (NS)
Day 1	5.36 ± 1.39	5.02 ± 1.64	0.317 (NS)
Day 2	3.86 ± 1.31	2.91 ± 1.28	<0.001 (S)
Day 3	1.96 ± 1.29	1.13 ± 0.89	0.002 (S)

## Discussion

Acute otitis media is the most common ENT condition, causing severe otalgia and affecting day-to-day activity and sleep disturbance in patients. Its diagnosis is mainly based on the signs and symptoms of the EAC inflammation, tragal tenderness, and edema [5]. The common pathogen causing acute otitis externa is *Staphylococcus aureus* [6]. Immediate intervention of pain in acute otitis externa is very important, as it is distressing for both patient and doctor [7]. The most effective treatment includes good aural toileting and medicated aural packs. As both gram-positive and gram-negative bacteria cause infections of the external ear, broad-spectrum antibiotics are the most effective treatment needed predominantly [8]. It is the most painful condition and needs gentle aural suctioning, topical drops, and oral analgesics. EAC edema can be reduced with IG packs, most commonly with cotton wicks. Various studies have used different materials for packing, and the results are varying [2,9]. Ichthammol is bacteriostatic and can slow down the growth of bacteria [10,11]. Adverse effects like skin irritation may occur [12]. Database research shows very few studies using polyvinyl alcohol (PVA) ear wicks in the past. Their use as a hemostatic plug in post-nasal surgeries is well documented in ENT, but not many studies have reported their use in the ear. One study done by Clamp shows merocele packs as an effective treatment for acute otitis externa when used with aqueous-based ear drops. In our study, we observed that the use of hydroxylated polyvinyl acetate medicated ear wicks and cotton wicks had similar efficacy based on senturia grading for mild to moderate cases. However, in severe cases, the cotton wick group showed better and faster recovery in terms of pain and edema compared to the PVA groups [13,14]. The probable reason could be that cotton provides better compression to the canal edema compared to expansile merocele packs and helps in reducing inflammation much faster [15]. PVA can be considered an alternative to cotton wicks in the treatment of acute otitis externa as they have many advantages, like coming in sterile packs, being able to be kept in the EAC for a longer duration, being easy to insert, and the risk of infection being comparatively lesser. Cotton wicks are used in most clinical settings as they are easily available, while PVA is not as easily available.

## Conclusions

Acute otitis externa is the most common clinical ENT condition, requiring urgent attention from the patient as it is a highly painful condition. Good local therapy by medicated wicks provides immediate symptomatic relief to patients. Cotton wicks have been routinely used for years in the treatment of acute otitis externa. An effective alternative to cotton wicks, PVA can be considered a highly advantageous material, producing nearly similar results to those of cotton wicks.
